# Autism Detection in Children by Combined Use of Gaze Preference and the M-CHAT-R in a Resource-Scarce Setting

**DOI:** 10.1007/s10803-021-04878-0

**Published:** 2021-02-16

**Authors:** Kelly Jensen, Sassan Noazin, Leandra Bitterfeld, Andrea Carcelen, Natalia I. Vargas-Cuentas, Daniela Hidalgo, Alejandra Valenzuela, Avid Roman-Gonzalez, Casey Krebs, Vincent Clement, Cody Nolan, Franklin Barrientos, Ardi Knobel Mendoza, Paola Noriega-Donis, Claudia Palacios, Andrea Ramirez, Macarena Vittet, Emil Hafeez, Mariana Torres-Viso, Myriam Velarde, Lawrence H. Moulton, Michael D. Powers, Robert H. Gilman, Mirko Zimic, On behalf of the Autism Working Group in Peru, Vanessa Cavallera, Ricardo Zavaleta, Juan Flores, Dennis Nuñez, Alejandro Dioses, Anna Smith

**Affiliations:** 1 Tulane University School of Medicine, New Orleans, LA USA; 2 School of Public Health and Tropical Medicine, Tulane University, New Orleans, LA USA; 3 Department of International Health, Johns Hopkins Bloomberg School of Public Health, Baltimore, MD USA; 4 Johns Hopkins University School of Nursing, Baltimore, MD USA; 5 Bioinformatics and Molecular Biology Laboratory, Research and Development Laboratory, Science and Philosophy Faculty, Universidad Peruana Cayetano Heredia, Lima, Peru; 6 Psychology Faculty, Universidad Peruana Cayetano Heredia, Lima, Peru; 7 Yale Child Study Center, Yale University School of Medicine, New Haven, CT USA; 8 Weill Cornell Medical College, New York, NY USA; 9 Rutgers Robert Wood Johnson Medical School, New Brunswick, NJ USA; 10 Johns Hopkins University Whiting School of Engineering, Baltimore, MD USA; 11 CCSN: The Center for Children With Special Needs, Glastonbury, CT USA; 12 IMLA: Instituto Medico Lengua Aprendazaje, Lima, Peru; 13 Present Address: Louisiana State University, Baton Rouge, USA; 14 Present Address: USA Indian Health Service, Albuquerque, USA

**Keywords:** Autism spectrum disorder, Developmental disorder, Eye tracking, Gaze preference, ASD diagnosis

## Abstract

Most children with autism spectrum disorder (ASD), in resource-limited settings (RLS), are diagnosed after the age of four. Our work confirmed and extended results of Pierce that eye tracking could discriminate between typically developing (TD) children and those with ASD. We demonstrated the initial 15 s was at least as discriminating as the entire video. We evaluated the GP-MCHAT-R, which combines the first 15 s of manually-coded gaze preference (GP) video with M-CHAT-R results on 73 TD children and 28 children with ASD, 36–99 months of age. The GP-MCHAT-R (AUC = 0.89 (95%CI: 0.82–0.95)), performed significantly better than the MCHAT-R (AUC = 0.78 (95%CI: 0.71–0.85)) and gaze preference (AUC = 0.76 (95%CI: 0.64–0.88)) alone. This tool may enable early screening for ASD in RLS.

**Supplementary Information:**

The online version contains supplementary material available at 10.1007/s10803-021-04878-0.

Autism spectrum disorder (ASD) encompasses a group of neurodevelopmental disorders characterized by variable deficits in communication that present in early childhood (American Psychiatric Association 2013; CDC 2016). According to the Centers for Disease Control and Prevention (CDC), one in 59 children in the United States is affected by ASD (Christensen et al. 2016). Although the prevalence of ASD has been increasing on a global scale, estimates of the worldwide prevalence are much lower, 1 in 160 children (Elsabbagh et al. 2012; WHO 2020) for several reasons. Among these are a lack of awareness of the disease both among physicians and the community overall, a lack of mental health infrastructure and practitioners and possible stigma regarding developmental delays, all of which lead to a lack of autism screening and detection (“Autism on the Rise” 2013; Christensen et al. 2016). The American Academy of Pediatrics recommends that children undergo screening for ASD at 18 months and 24 months (C. P. Johnson et al. 2007). Typically, a screening test, such as the Modified Checklist for Autism in Toddlers-Revised (M-CHAT-R) is used and is considered positive if two of the critical questions were positive and /or three or more of the non-critical questions were positive. This is followed by a diagnostic work-up to confirm and determine the disorder severity (CDC 2016; Robins et al. 2014). Diagnostic tests include the Autism Diagnostic Observation Schedule-II (ADOS-II), which requires observation of the child by a trained clinician and the Autism Diagnostic Interview Revised (ADI-R) in which the child’s parents are interviewed. These diagnostic tests together with a child’s overall developmental profile are used to generate a comprehensive clinical diagnosis of ASD (Le Couteur 2003). While the ADOS-II is often considered the “gold standard” evaluation procedures for the identification of ASD, it must be carried out by trained health professionals and is both time and resource intensive (Paula et al. 2011; Delfos 2011). As a result, its applicability and availability to low-resource settings is limited even as the need exists in such settings for screening tools for ASD.

Numerous studies demonstrate improved intellectual and adaptive behavior in children with early intervention utilizing the broad principles and practices of Applied Behavior Analysis (ABA) (Estes et al. 2015; Helt et al. 2008; Reichow et al. 2012; Warren et al. 2011). However, in the absence of widespread and evidence-based screening and diagnostic evaluation procedures, access to otherwise valuable early intervention opportunities is substantially compromised.

The aim of this study was to investigate the validity of a portable gaze-preference system as a low-cost screening tool for ASD and demonstrate consistency of the screening tool at follow-up study visits. Though previous studies on eye tracking for autism diagnosis exist, the software and protocols are expensive, and require specialized equipment thus limiting their applicability. Our a priori hypothesis was that the concurrent use of our low-cost gaze preference system and the MCHAT-R questionnaire as a joint screening tool is able to predict ASD status. Initial examination of our data led to the following a posteriori hypotheses: (a) the joint screening tool can be used for better specificity, (b) the initial 15 s of the gaze-preference video is at least as predictive as the entire video and (c) the duration of gaze on the social scene is more predictive than that on the abstract scene.

## Methods

### Study Design and Participants

Children 36–99 months old were recruited from educational centers throughout Lima, Peru. Children with ASD were identified through three private child development centers in Lima, including the Instituto Medico Lenguaje y Aprendazaje, (IMLA), where they were receiving therapy for a previously established diagnosis of ASD. The diagnosis of ASD for these children was consistent with criteria from the Diagnostic and Statistical Manual of Mental Disorders-5^th^ Edition (American Psychiatric Association 2013), based on a combination of clinical evaluation and previous exams including evoked auditory potentials, language and sensorial processing evaluations, and psychological evaluations. Participants were excluded if they had a primary developmental disability diagnosis other than ASD, such as a language disorder, general developmental disorder, or learning disability, in addition to autism. The control group consisted of typically developing (TD) children who were identified through public schools and daycare centers in Lima, Peru. Control-group children were eligible if they had no previous diagnosis of ASD, specific language disorder, general developmental disorder, learning disability, intellectual disability, or neurologic illness, and no illness at the time of enrollment.

The MCHAT-R was selected for use as a component of the screening protocol because of its broad availability in a number of languages including Spanish and its ease and accessibility of administration (Canal-Bedia et al. 2011; M-CHAT™—MCHAT R/F Translations 2020). While the MCHAT-R is widely used as a screener for possible ASD (Coury et al. 2017) it is not without its limitations. In addition, traditionally the MCHAT-R is considered most valid as a screening tool for children through 48 months of age. However, given that children in Peru do not typically present for, and are not usually identified with, ASD until 36 months or later (Observatorio Nacional de la Discapacidad | CONADIS Peru—OBSERVATORIO DE LA DISCAPACIDAD 2020), we elected to expand the study group age range to 99 months in order to access a larger pool of individuals for this exploratory study.

All children were screened with the MCHAT-R- and ADOS-II at the time of recruitment.

Control-group children who screened positive with the ADOS-II evaluation at the time of enrollment were excluded from analysis, as they lacked a clinical diagnosis of ASD.

### Gaze Preference Recording

We used a standard laptop with a 17-inch screen with two videos, one of social scenes and the other of abstract scenes playing, separated by 8 inches (Vargas-Cuentas et al. 2017). The original video used for the testing, was saved with a resolution of 1920 × 1080 pixels, at an aspect ratio of 16:9, and displaying at 60 frames per second. The social and the abstract images displayed were projected in rectangular areas on the left and on the right of the screen. The size of the image was 37.5% of the width and 37.04% of the height of the screen. The original video was recorded at a temporal resolution of 60 frames per second and this is the way it is “projected” in the screen. However, the camera that recorded the video of the face of the child, captured a video with a temporal resolution of 30 frames per second.

For the gaze preference screening, each child watched a minute-long video on a computer with a front-mounted camera that recorded their face movements. The video consisted of a 10-s introductory scene to attract the child’s attention, followed by a split screen showing the abstract scene on the right side of the screen and the social scene shown on the left. The social scene consisted of a video of young children playing and interacting with each other, and the abstract scene consisted of moving shapes of various colors. These visits took place at the respective recruitment sites. Each child was seated in a chair 30 cm from the computer screen. If the child was unable to sit alone, they were placed in their guardian’s lap 30 cm from the computer screen. This distance allowed for clear visibility of the eyes by coders without being uncomfortable for the child. If the child’s attention turned away from the video, they were redirected either with auditory or visual cues to the screen by their guardian or supervising study personnel. The procedure was repeated approximately one month after the initial recording. No adverse effects were observed associated with this procedure.

### Gaze Preference Coding

The gaze-preference device was based on the study by Vargas-Cuentas et al. 2017. The current study was done to provide the proof-of-concept for the validity of our approach and correctness of our hypotheses. We used manual coding to bypass any coding software issues. We are in the process of improving our coding software to enable fully automated screening process. Study personnel reviewed each video at a rate of 2 frames per second, one for every 15 frames filmed (101 frames per child). At each time point the child’s gaze was labeled as either left, right, center or distracted. Left indicated focus on the social scene, right indicated focus on the abstract scene. Distraction was defined as any time that the child was not looking within the frame of the video or their eyes were not visible. Center was when the child was looking at the center of the screen between the social and abstract video. Two separate, independent groups of trained coders who were blinded to the diagnostic status of the subjects coded the videos taken at visit one and visit two. In cases of discrepancy, the two coders reanalyzed the frame in question and decided the gaze direction with consensus. Given the constraints of the study conditions, a more traditional method of determining interrater reliability was not undertaken. Gaze preference was determined by calculating the proportion of the stimulus display time—or equivalently, the proportion of all frames—the child spent looking at the social scene, abstract scene, center of the screen, or not looking at the screen (distraction time).

### Statistical Analysis

Stata 14.2, StataCorp, College Station, Texas, USA was used for statistical analysis. To demonstrate the gaze-preference patterns among ASD and typically developing (TD) children over the course of the video, we used LOWESS smoothing (locally weighted scatterplot smoothing) (Cleveland and Devlin 1988) with a bandwidth of 0.8. A Mann–Whitney Test was used to compare proportions of social and abstract scenes and distraction times between TD children and children with ASD. We verified the consistency of these results between two separate gaze-preference visits by intra-class correlation ICC) between visits. We employed Wilcoxon signed-rank test to compare gaze preference of the same group of children at different segments of the video. To develop a two-step approach to classifying ASD and control children, we estimated a logistic regression model and used the Hosmer–Lemeshow test with group sizes of 5 and 10 in separate tests to examine the model goodness of fit. We also report the chi-sq test of goodness of fit between the predicted and actual ASD status and have included a plot of their predicted and actual values and a plot of predicted probability vs the number of social scenes in the supplement. This model used the results of the MCHAT-R in addition to gaze preference as predictors of ASD status. We used AUC to compare models based on the first 15 s of gaze-preference data against one based on the entire 50 s length of the video and subsequently we tested the model in one half of the sample and used each to calculate the AUC for the other half of the sample. Additionally, we tested the coefficient of the interaction term between the dummy variable indicating each half of the sample and each of the main model predictors (MCHAT-R and gaze preference results) to test whether the models from the two halves of the sample were different. We used a similar approach to test whether a model based on the age groups shared by cases and controls could predict the ASD status of children outside those age ranges. We also computed sensitivity of the model at specificity levels of approximately 70%, 80% and 90% to indicate how model sensitivity would change at different levels of specificity. To study the association between children’s ADOS-II scores and their gaze preference, the Mann–Whitney test was used. Participating children were excluded from the analysis if they were controls with a positive ADOS-II score (n = 7) in which case their true ASD status could not be ascertained or if their MCHAT-R or ADOS-II scores were missing (n = 16). Additionally, there were two controls and one case excluded due to acute illness.

## Results

### Focus Area Discrimination Between ASD and TD Groups

We administered the gaze-preference test at 2 separate occasions, on average 1 month apart. The analyzable sample size in visits one and two were 101 (73 TD controls and 28 children with ASD) and 100 (73 controls and 27 children with ASD), respectively. Cases were predominantly male (89%) and older (median = 60 months) compared to controls who were 51% male and younger (median = 42 months) (Table 1). Please also see tables S.1 in the Supplement.

**Table 1:** Age, sex and ADOS-II score distribution in TD and ASD diagnosed children

	N	Age				ADOS-II			
		mean	sd	min	max	mean	sd	min	max
TD									
F	37 (51%)	42.6	4.5	36	51	1.6	2	0	7
M	36 (49%)	43.0	4.7	36	50	0.5	1.4	0	7
ASD									
F	3 (11%)	48.0	12	36	60	22.7	4.9	17	26
M	25 (89%)	61.3	14.5	36	99	18.6	5.8	6	26

**Fig. 1:**
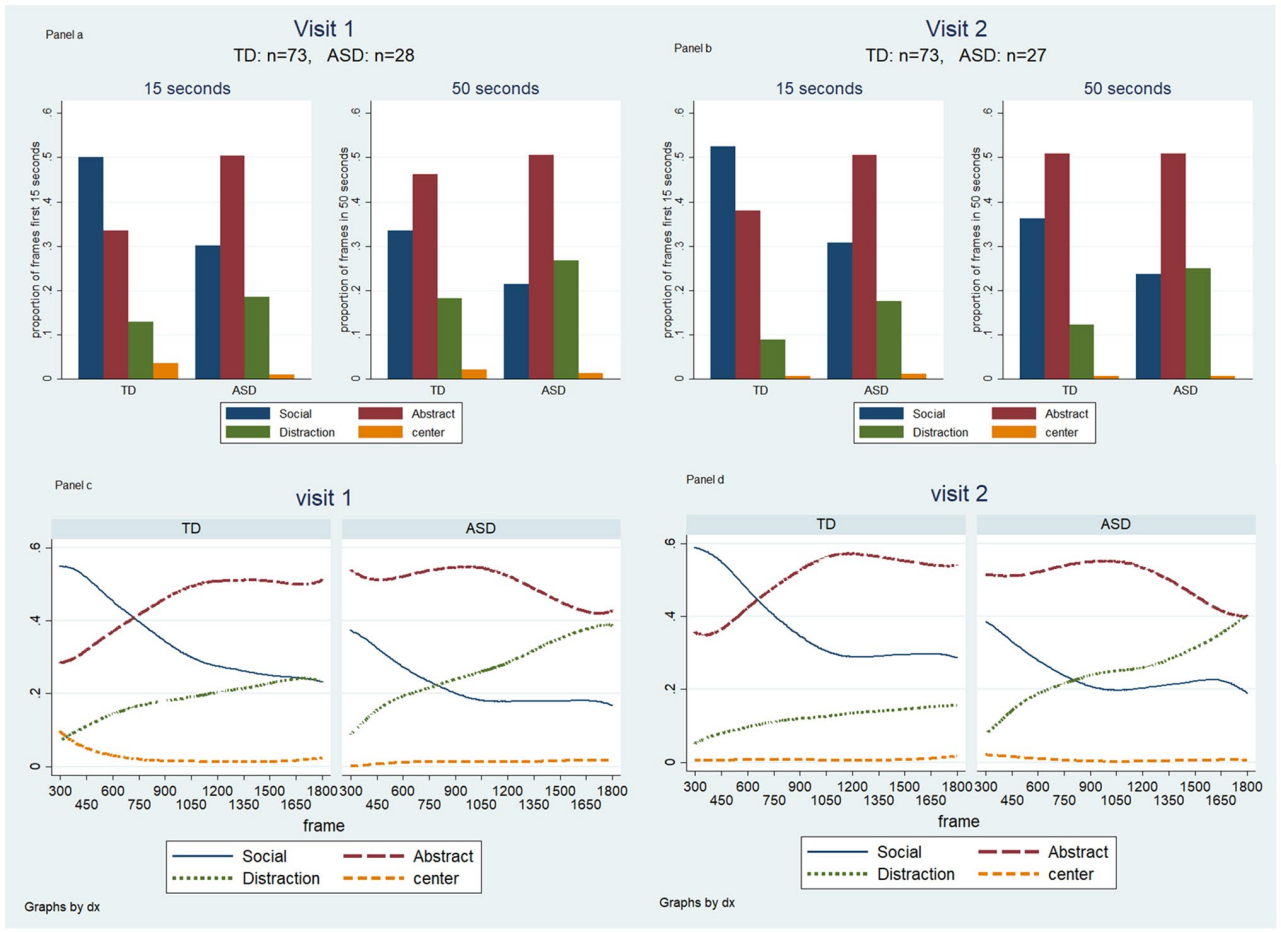
**a** and **b**: Focus areas of TD children and children with ASD during the first 15 s and the full 50 s at visit 1 **a** & visit 2 **b**. Each cluster of bars totals 100%. **c** and **d**: Focus trends among the two groups in the 50 s video in visit 1 **c** & visit 2 **d**. In **c** and **d**, LOWESS lines demonstrate the smoothed proportion of gaze at each scene by cases and controls at every ½ second of the video, depicting change in gaze direction as time elapsed over the duration of the video

Gaze preference classification detected differences between the group of children with ASD and the TD control group (Table 2 depicts these differences in visit 1 while visit 2 results are presented in table S.3 in the Supplement). As seen in Table 2, over the full 50 s, children with ASD spent significantly less time than the TD control group focused on the social scene at both visit 1 (21.5% vs 33.5% of the length of the video, p < 0.0001) and visit 2 (24% vs. 36%, respectively, p < 0.001). Furthermore, although children with ASD were not significantly more distracted than TD children in the first visit (26.7% vs 18.3%, p = 0.170) (Table 2; Fig. 1a, b) this difference was significant in the second visit (25.0% vs 12.2%, p = 0.013). By contrast, attention to the abstract scene during the 50 s, although higher, was not significantly so, among ASD-diagnosed children in visit 1 (50.5% vs 46.2%, p = 0.358) or visit 2 (50.8% vs 50.9%, p = 0.920). Gazing at the center of the screen was rare and it was inconsistent between the two visits among TD children. (Table 2 and Fig. 1a and b, for more detailed graphs and LOWESS with different bandwidth see Supplement Figures S.4-S.6).

**Table 2:** Gaze-preference behavior, visit 1: table entries indicate the proportion (SD) of all frames gazed at. (Visit 2 is presented in the Supplement)

Time course	Focus area	ASD mean (SD)	TD mean (SD)	Significance
First 15 s (30 frames)	Social	0.301 (0.215)	0.501 (0.196)	P < 0.001
	Abstract	0.503 (0.238)	0.335 (0.176)	P = 0.001
	Center	0.010 (0.027)	0.035 (0.045)	P = 0.002
	Distracted	0.185 (0.217)	0.129 (0.149)	P = 0.175
First 30 s (60 frames)	Social	0.237 (0.165)	0.398 (0.174)	P < 0.001
	Abstract	0.542 (0.237)	0.419 (0.172)	P = 0.013
	Center	0.011 (0.025)	0.026 (0.037)	P = 0.003
	Distracted	0.210 (0.200)	0.157 (0.133)	P = 0.421
50 s (100 frames)	Social	0.215 (0.163)	0.335 (0.154)	P < 0.001
	Abstract	0.505 (0.227)	0.462 (0.167)	P = 0.358
	Center	0.013 (0.019)	0.021 (0.019)	P = 0.049
	Distracted	0.267 (0.218)	0.183 (0.129)	P = 0.170

Viewing the social scene, the abstract scene, the center of the screen or being distracted during the first 15, first 30 or the entire 50 s of the video in visit 1. Example: Children with ASD and TD children, respectively, gazed at the social scene 30.1% and 50.1% of all frames during the first 15 s of the video

### Time Course Analysis

Graphs for visit 1 and visit 2 (Fig. 1a–d) suggest a very high degree of consistency in gaze patterns between the two visits (based on the full length of the video, the between visit ICC for social scene = 0.54, 95%CI: 0.40–0.68, for abstract scene = 0.44, 95%CI: 0.29–0.60, for distraction = 0.41, 95%CI: 0.24–0.57, for center = 0, 95%CI: 0–0.20).

Visual inspection of graphs of smoothed trajectories of gaze preference behavior (Fig. 1c and d) suggests that the first 15 s maximally discriminate between children with ASD and controls in both visits: as most TD children started the video by gazing at the social scene an increasingly fewer proportion of them did so until the trajectory for social scene viewing crossed that for abstract scene at around the 600^th^ frame (17^th^ second). By contrast, children with ASD tended to view the abstract scene throughout this segment and paid increasingly less attention to the social scene. Based on these observations, we hypothesized that gaze patterns during the first 15 s of the video discriminated between children with ASD and TD controls as well as gaze patterns over the entire length (50 s) of the video (Fig. 1a and b and Table 2).

Attention to the social scene diminished among both TD children and those with ASD as time elapsed (Wilcoxon signed rank test of difference between 15 and 50 s: 50.1% vs 33.5%, p < 0.001 for control group, 30.1% vs 21.5%, p < 0.001 for ASD group) while distraction increased in both groups (Wilcoxon signed rank test of difference between 15 and 50 s: 12.9% vs 18.3%, p < 0.001 for control group, 18.5% vs 26.7%, p < 0.041 for ASD group). Children with ASD spent about 50% of their time viewing the abstract scene, and this tendency did not change significantly throughout the video (Wilcoxon signed rank test of difference between 15 and 50 s: 50.3% vs 50.5%, p = 0.793). By contrast, typically developing children paid significantly less attention to the abstract scene during the first 15 s of the video but paid more attention as time elapsed (Wilcoxon signed rank test: 33.5% vs 46.2%, p < 0.001*).* These patterns and the significant differences reported in Table 2 suggest that the data from the first 15 s of the video are as discriminating between the ASD and control groups as the data from the entire length of the video for social focus and distraction, while abstract scene viewing is a significant discriminator only in the first 15 s.

### Association of the First 15 s of Gaze-Preference Behavior with ADOS-II

**Fig. 2:**
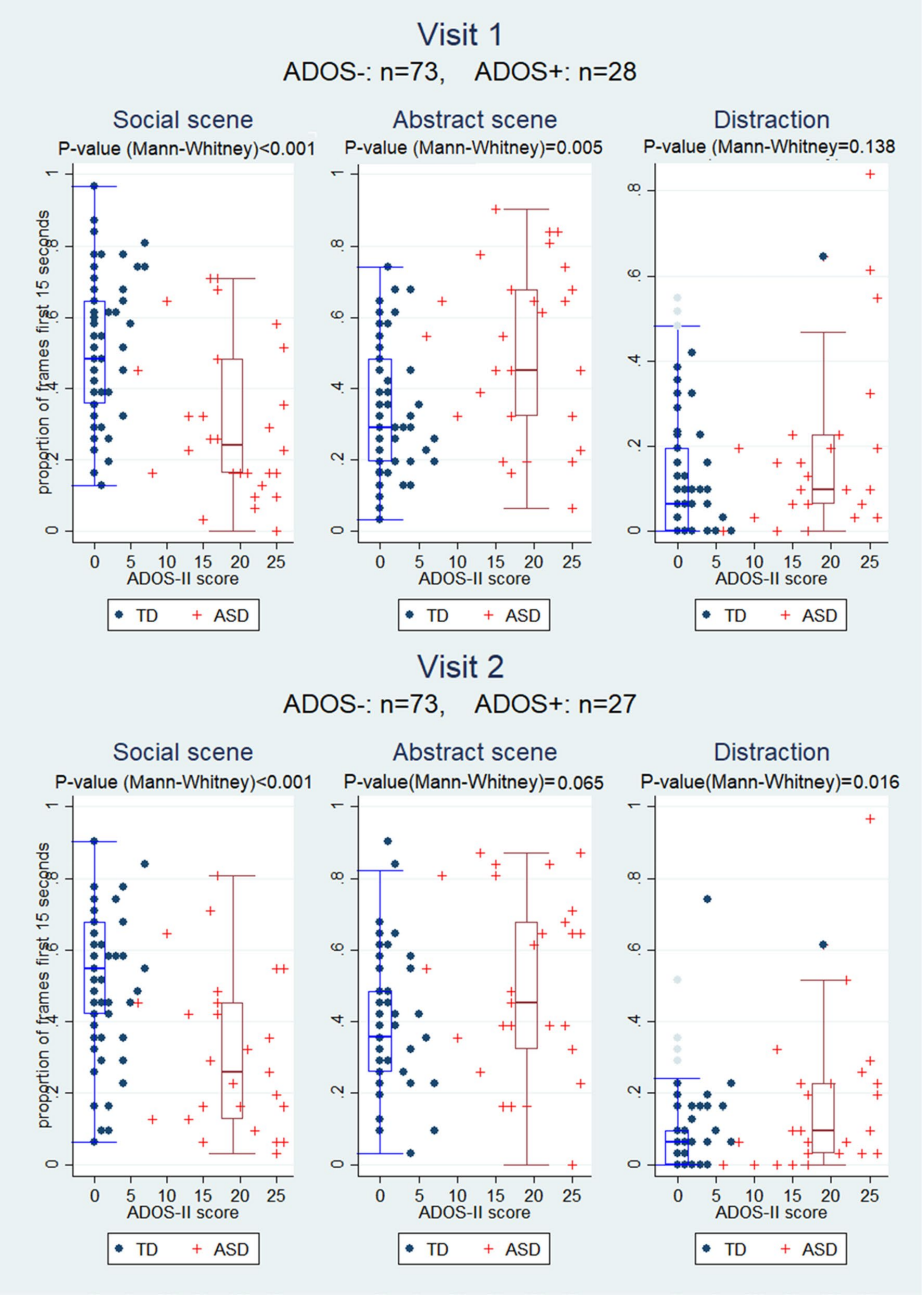
Association of gaze-preference behavior during the first 15 s with established ADOS-II diagnosis

Figure 2, corresponding with the first and the second visit, demonstrates that ADOS-II classification was inversely associated with the proportion of social scene frames during the first 15 s; i.e., ADOS-II-positive children viewed the social frame less frequently (Fig. 2). By contrast, ADOS-II classification was directly associated with the proportion of abstract scene frames suggesting that ADOS-II positive children preferred the abstract scene. We also observed that distraction during the first 15 s of the video was higher in ADOS-II-positive children. These associations were statistically significant.

### The Two-Step Approach to Modeling Autism Using Gaze Preference and the MCHAT-R: Development of the GP-MCHAT-R Model

**Fig. 3:**
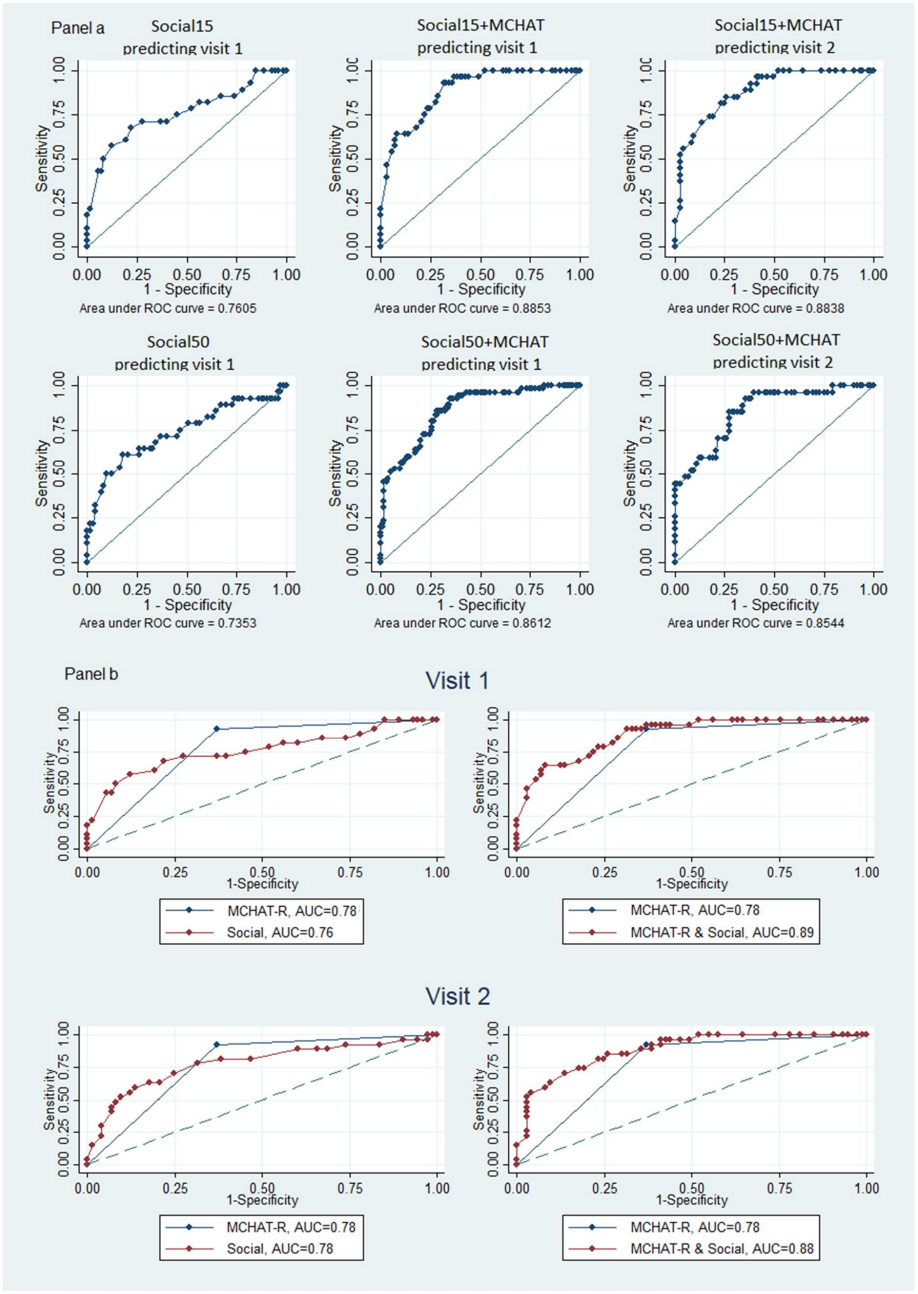
**a** ROC curves and AUC’s based on logistic models, first 15 s and the full 50 s. Models were developed using visit 1 data and were used to classify visit 1 and visit 2 data. Model predictors: Social15 represents the amount of time spent viewing the social scene during the first 15 s of the gaze preference data. Social50 represents the amount of time spent viewing the social scene during 50 s of the gaze-preference data. Social15 + MCHAT represents the amount of time spent viewing the social scene during the first 15 s and the MCHAT combined. **b** predictability of ASD based on attention to the social scene in the first 15 s alone, MCHAT-R alone or the GP-MCHAT-R model, compares the ROC curves and AUC values for the models based on the first 15 s. The left panels compare MCHAT-R against social scene (first 15 s) prediction. The right-side panels compare MCHAT-R against the 2-step model prediction

Gaze preference over the full length of the video could discriminate between TD children and those with ASD, but we also observed that the initial 15 s of the video was at least as discriminating which prompted us to hypothesize that a shorter video could represent gaze preference before distraction sets in. This was also suggested by Fig. 1, by the steadily rising distraction lines. We hypothesized that the first few seconds before the lines for abstract and social scene cross and when the difference between the proportion of abstract and social gazes are almost opposite (on average) between cases and controls (prior to frame 600) and while distraction is at its lowest, we could get the most discriminating information. A predictive logistic model with one or more gaze-preference variables also suggested this (Table 3ai). In our sample, MCHAT-R with critical questions demonstrated a sensitivity of 93% (95% CI: 76–99%) and a specificity of 63% (95% CI: 51–74%), with an AUC of 0.78. We used a 2-step screening including gaze preference and MCHAT-R to improve on the MCHAT-R’s low specificity while incorporating its high sensitivity. Of the gaze-preference variables, social scene focus was the most predictive option, and we used it in combination with MCHAT-R results to construct a parsimonious, unweighted logistic regression model, the GP-MCHAT-R model (Table 3ai, ii). Adding other gaze preference variables within the same model did not improve the fit or model prediction. We also examined a model with both abstract scene and distraction – but without the social scene – which could predict ASD similarly well, but we used the model with social scene since it was more parsimonious. The Hosmer–Lemeshow test for the model based on social scene indicated good fit throughout the response range (p = 0.397 based on quintiles of data and p = 0.577 based on deciles). In the Supplement, we have presented the details of the Hosmer–Lemeshow test as well as two graphs presenting a histogram of model predicted probabilities in the two groups of children and the predicted probabilities plotted against social15 values (tables S.4 and S.5 and graphs S.1 and S.2). In another step for model validation, we initially estimated the model in each of two random halves of the sample (seed = 99, n1 = 55 and n2 = 46) and produced the ROC curve for the estimation half (AUC1 = 0.89 and AUC2 = 0.87) as well as the validation half (AUC3 = 0.88 and AUC4 = 0.89). To ensure the model applicability to different random subsamples of the data, we conducted an analysis to test the coefficient of the interaction terms between the dummy variable representing the two random subsamples separately with social15 and MCHAT-R in the model. The p-values for these interactions (0.681 and 0.622, respectively) suggest no difference in these coefficients when estimated in each subsample. Results are presented in the Supplement (Table S.6 and Figure S.3). Table 3a and Fig. 3a demonstrate the 15-s model and the 50-s model. To verify this, we conducted a likelihood-ratio test to examine the effect of the presence or absence of each of social15 and social50 in a model with MCHAT-R. The full model consisted of MCHAT-R, social15 and social50 as predictors. We tested this against a reduced model with social15 but without social50 and one with social50 but without social15 (likelihood-ratio test comparing each reduced model against the full model: p = 0.464 and 0.009, respectively) and concluded that the absence of social15 in the model leads to a significantly worse model whereas absence of social50 is not a significant detriment.

**Table 3:** Logistic regression models and their associated sensitivity, specificity and AUC

A. Predictive Models (Visit 1)	Predictors	OR	P	95% Conf. Int	
i. First 15 s (GP-MCHAT-R model)	Social-15 s	0.85	0.001	0.78	0.94
Model LR = − 37.96, AIC = 0.811	MCHAT-R	21.6	< 0.001	4.5	103.83
	constant	0.32	0.198	0.06	1.82
ii. Full 50 s	Social-50 s	0.96	0.013	0.92	0.99
Model LR = − 41.06, AIC = 0.873	MCHAT-R	19.83	< 0.001	4.26	92.25
	constant	0.17	0.04	0.03	0.92

B. Sensitivity of GP-MCHA-R model at approx 90%, 80% and 70% specificity	Model AUC	Specificity approx 90%		Specificity approx 80%		Specificity approx 70%	
		*spec*	*sens*	*spec*	*sens*	*spec*	*sens*
Visit 1 Visit 2	0.89 0.88	92% 90%	64% 63%	78% 77%	75% 81%	69% 70%	93% 85%

Because the age distribution of the children with ASD and TD controls were different (see Tables S.1 and S.2 in the Supplement) we fitted the model with social15 and MCHAT-R but included a dummy variable identifying ages that were common to both children with ASD and TD children (45–49 months). We then fitted a logistic model with social15, MCHAT-R, the dummy variable and the interaction terms, similar to what was described above for the random subsample analysis (this is also discussed in the Supplement: Comparing the GP-MCHAT-R Model in the Age Groups Including Both Cases and Controls Against Other Ages). Results suggested that the coefficients for social15 and MCHAT-R were applicable to both age groups (p-value for social15 = 0.781, p-value for MCHAT-R = 0.332).

Figure 3a depicts the ROC curves and associated AUC’s for both models in visit 1 and visit 2 data (AUC values are 0.89 and 0.86 for the 15- and the 50-s models). As Fig. 3b indicates, although the AUC for gaze preference (social scene focus in the first 15 s) is slightly less than that for MCHAT-R alone (0.76 vs 0.78, p = 0.796), the combined AUC for the 2-step model, which includes both MCHAT-R and social scene as predictors, presents a significant improvement over MCHAT-R alone (0.89 vs 0.78, p < 0.001), particularly on model specificity.

Considering the importance of both sensitivity and specificity for an effective screening tool and the tendency of MCHAT-R to give false alarms, we also assessed the GP-MCHAT-R model’s sensitivity and specificity in addition to its AUC. Table 3bi demonstrates the sensitivity of the GP-MCHAT-R model associated with specificities of approximately 90%, 80% or 70% in each of visit 1 and visit 2. The GP-MCHAT-R model AUC in visit 1 is 0.89 and in visit 2 it is 0.88 (Fig. 3a). This model has a sensitivity of 64% at a specificity level of exactly 92% when applied to visit 1 data, and a sensitivity of 63% given a specificity of 90% when applied to visit 2 data. Thus, the model can provide an alternative to the low specificity of MCHAT-R when a lower sensitivity is acceptable.

## Discussion

This study demonstrates that combining gaze preference, as psychophysical behavioral coding of preferential looking in infants and toddlers, using data from a 15-s initial observation period and the MCHAT-R provides better sensitivity and specificity than either test alone. Recently, eye-tracking studies in ASD have included examining visual saccades, speed and accuracy of eye movements, and differences in eye fixation regions on human faces (Chawarska et al. 2016; Harrop et al. 2018; B. P. Johnson et al. 2016; Jones and Klin 2013; Klin et al. 2009; Kovarski et al. 2019; Mottron et al. 2007; Pierce et al. 2011, 2016; Vargas-Cuentas et al. 2017). Overall, our application is consistent with findings from prior gaze preference studies.

Our principal finding was that gaze preference could be a basis for community surveillance of ASD and also that MCHAT-R and gaze-preference results could be combined in a predictive model.

Our work confirmed the results previously described by Pierce et al. (Pierce et al. 2011, 2016) that gaze preference over the full length of the video could discriminate between TD children and those with ASD, but we also observed in our data that the initial 15 s of the video was somewhat more discriminating. Therefore, we hypothesized that a shorter video could represent gaze preference before distraction sets in. This was also suggested by the LOWESS graphs, by the steadily rising distraction lines. Our hypothesis based on this observation was that the first few seconds before the lines for abstract and social scene cross and when the difference between the proportion of abstract and social gazes are almost opposite (on average) between cases and controls (prior to frame 600, the 17th second of the video) and while distraction is at its lowest, we could get the most discriminating information.

Within the first 15 s of the videos, we observed that TD children spent significantly more time focusing on the social scene than the abstract scene while for the children with ASD the reverse was true. Later in the video, TD children’s attention shifted from the social scene to the abstract scene while those with ASD paid somewhat less attention to the abstract scene and were increasingly distracted but did not shift to the social scene. A shorter video has the advantage of reducing children’s fatigue and loss of interest and increased ease of coding. Therefore, we developed our index screening tool, GP-MCHAT-R using gaze preference during the first 15 s of the video augmented with the MCHAT-R classification results, to predict the risk of ASD with more specificity than either test alone.

Considering the importance of both sensitivity and specificity for an effective screening tool, we chose a model with increased specificity to identify children who may be at increased risk of ASD while minimizing false positives in order to improve on the poor specificity of the MCHAT-R. The GP-MCHAT-R model can be adjusted to focus on either specificity or sensitivity, depending on the need of the model. In low-prevalence, resource limited settings, it can be applied with a highly specific cutoff with a sensitivity of 70% to decrease the number of false positives but with an increase in false negatives. In low-resource settings the increase in specificity decreases the burden on the already limited number of expert clinicians diagnosing ASD.

While there has been an increased focus on gaze-preference differences in ASD, to our knowledge no study has developed a simple, portable gaze-preference screening device. Previous studies have been conducted in a research laboratory, using special equipment and high-power computer software (Jones and Klin 2013; Klin et al. 2009; Kovarski et al. 2019; Mottron et al. 2007). These factors may pose a significant barrier in countries like Peru, which has a widely dispersed rural population and an urban population with difficulties accessing transportation outside of their neighborhood. Thus, families may be unaware of the challenges their child is exhibiting, unaware of screening resources, or may confront stigma associated with ASD. These issues argue strongly for the importance and availability of portable, locally-sourced and delivered screening tools that can be reliably administered by community-based healthcare workers and providers. The application described in this study can be used on any computer with a built-in or mountable camera and data can be collected in as little as 15 s by a trained community healthcare worker.

There are a number of limitations to this study, many due to setting and access variables noted earlier. Recognizing that in Peru children do not typically present for evaluation of ASD earlier in toddlerhood, we extended the accepted MCHAT-R age range of study participants beyond the typical 48-month cutoff in order to access sufficient children. We deemed this appropriate in order to validate the combined GP-MCHAT-R screening protocol and to investigate “proof-of-concept”. Other limitations include the lack of age matching and differences in baseline demographics. Cases were predominantly male and on average over one year older than controls. This was largely unavoidable, considering that autism is more prevalent in males and access to autism screening for toddlers in Peru is limited. Nonetheless, it led to a very small number of girls in the ASD group. Other limitations of the study were the unknown treatment history of children with ASD prior to our study and time from clinical diagnosis of children with ASD to the first gaze preference test. Additionally, we did not evaluate the cognitive abilities of the participants and did not collect socio-economic information other than sex and age. As mentioned earlier in Gaze Preference Coding, given the constraints of the study conditions, a more traditional method of determining interrater reliability was not undertaken. This is an important limitation of the study, although we attempted to address this by having the two trained coders resolve any discrepancies in coding child behavior while reviewing the tapes. While not an ideal method, we determined that financial and setting constraints of the study necessitated this less strenuous approach. It is equally important to note that this work is preliminary in nature, was designed to investigate the viability of a simple and straightforward screening system that could be used in resource-scarce areas, in the service of establishing parameters for future investigations of the screening tool that we developed. Finally, due to study limitations we did not include a second control group consisting of children with developmental delays who did not meet diagnostic criteria for ASD. In order to be maximally useful, a screening measure must demonstrate adequate specificity in order to discriminate between children with ASD from those with other developmental challenges. There are several important reasons for this, including in the present case ensuring that scarce human and financial resources are allocated to the children who need them the most. Recognizing that the M-CHAT-R alone may overidentify children with other problems as being at higher risk for ASD, we combined a more robust and well-validated tool—the preferential selection of nonsocial over social stimuli by children with ASD—as an adjunctive strategy for screening, effectively integrating the two tools into a single screening concept. While this does not minimize the importance of comparing and validating the method described in this paper with a follow-up sample of children with ASD, children with an established neurodevelopmental disorder without ASD, and a typically developing sample, we also highlight the exploratory nature of the present work, seeking as it did to determine the efficacy of combining gaze preference with the M-CHAT-R. Our ongoing investigations will address and further refine valid concerns regarding the specificity of the proposed screening tool.

Each of these limitations should be considered and addressed in future efforts to evaluate the viability of the GP-MCHAT-R screener.

The combined GP-MCHAT-R model was designed as an enhanced screening tool and should not be considered a replacement for clinical diagnosis. Rather, a positive screen with the GP-MCHAT-R should guide healthcare professionals to a referral for a more comprehensive ASD diagnostic workup.

In conclusion, we demonstrate that visual preference—both social focus and distraction time—serve as markers for increased risk of ASD in toddlers. Differences are detectable within as little as fifteen seconds of gaze-preference data and combined with the MCHAT-R (GP-MCHAT-R) produces a high degree of reliability with improved sensitivity and specificity, compared with the MCHAT-R alone. Use of the GP-MCHAT-R model has the potential to decrease barriers to early ASD screening in resource-limited settings, ultimately enhancing the potential of better outcomes for toddlers with ASD.

## Supplementary Information

Below is the link to the electronic supplementary material.

Supplementary file1 (docx 222 KB)

## Abbreviations

Gp: Gaze Preference
ET: Eye tracking
ADOS-II: Autism Diagnostic Observation Schedule-II
GP-MCHAT-R: Gaze preference combined with the MCHAT-R for ASD detection
ASD: Autism spectrum disorder
TD: Typically developing children
MCHAT-R: Modified Checklist for Autism in Toddlers-Revised
ABA: Applied Behavior Analysis
ADI-R: Autism Diagnostic Interview Revised
LOWESS: Locally weighted scatterplot smoothing
ROC: Receiver-operating curves
AUC: Area under the ROC curve
