# Supplementary material for: Autism Detection in Children by Combined Use of Gaze Preference and the M-CHAT-R in a Resource-Scarce Setting
Source: J Autism Dev Disord. 2021 Feb 16;51(3):994–1006. doi: 10.1007/s10803-021-04878-0 (PMC7954728; doi:10.1007/s10803-021-04878-0)

**Autism Detection in Children by Combined Use of Gaze Preference and the M-CHAT-R in a Resource-Scarce Setting**

**SUPPLEMENT**

***Age Distribution in Cases and Controls***

**Table S.1**

Age Distribution

| Age (months) | TD | ASD | Total |
| --- | --- | --- | --- |
| 36 | 12 | 2 | 14 |
| 39 | 6 | 0 | 6 |
| 40 | 7 | 0 | 7 |
| 41 | 8 | 0 | 8 |
| 42 | 6 | 0 | 6 |
| 43 | 5 | 0 | 5 |
| 44 | 1 | 0 | 1 |
| 45 | 3 | 1 | 4 |
| 46 | 4 | 1 | 5 |
| 47 | 2 | 0 | 2 |
| 48 | 10 | 3 | 13 |
| 49 | 5 | 1 | 6 |
| 50 | 3 | 0 | 3 |
| 51 | 1 | 0 | 1 |
| 54 | 0 | 1 | 1 |
| 56 | 0 | 2 | 2 |
| 60 | 0 | 9 | 9 |
| 63 | 0 | 2 | 2 |
| 72 | 0 | 3 | 3 |
| 78 | 0 | 1 | 1 |
| 96 | 0 | 1 | 1 |
| 99 | 0 | 1 | 1 |
| Total | 73 | 28 | 101 |

***Number of Frames of Social15 by Age (social scene the first 15 seconds)***

**Table S.2**

Number of social15 frames among children in age groups common to cases and controls (45-49 months) and among other ages in visit 1 and visit 2

|  | Ages 45-49 months | | | <45 months | |  | >49 months | |  |
| --- | --- | --- | --- | --- | --- | --- | --- | --- | --- |
|  | mean | median | N | mean | median | N | mean | median | N |
| **visit 1** |  |  |  |  |  |  |  |  |  |
| TD | 15.2 | 16 | 24 | 15.5 | 15 | 45 | 17.8 | 17 | 4 |
| ASD | 8.5 | 6.5 | 6 | 9.5 | 9.5 | 2 | 9.6 | 7.5 | 20 |
| **visit 2** |  |  |  |  |  |  |  |  |  |
| TD | 15.9 | 17 | 24 | 16.5 | 17 | 45 | 15.8 | 16 | 4 |
| ASD | 9.2 | 7.5 | 6 | 15 | 15 | 1 | 9.4 | 7.5 | 20 |

***Gaze Preference Over the Length of the Video (visit 2)***

**Table S.3**

Gaze-preference behavior, visit 2: table entries indicate the proportion (SD) of all frames gazed at

| **Time Course** | **Focus Area** | **ASD Mean (SD)** | **TD Mean (SD)** | **Significance** |
| --- | --- | --- | --- | --- |
| First 15 seconds (30 frames) | Social | 0.307  (0.2018) | 0.524  (0.178) | P < 0.001 |
|  | Abstract | 0.505  (0.254) | 0.380 (0.166) | P = 0.020 |
|  | Center | 0.012 (0.022) | 0.006 (0.018) | P = 0.079 |
|  | Distracted | 0.176 (0.222) | 0.089 (0.125) | P = 0.037 |
| First 30 seconds (60 frames) | Social | 0.241 (0.183) | 0.409 (0.157) | P < 0.001 |
|  | Abstract | 0.544 (0.232) | 0.478 (0.167) | P = 0.250 |
|  | Center | 0.007  (0.011) | 0.006 (0.016) | P = 0.263 |
|  | Distracted | 0.208 (0.227) | 0.108 (0.104) | P = 0.067 |
| 50 seconds (100 frames) | Social | 0.236  (0.179) | 0.362  (0.145) | P < 0.001 |
|  | Abstract | 0.508 (0.225) | 0.509 (0.156) | P = 0.920 |
|  | Center | 0.006 (0.010) | 0.006 (0.015) | P = 0.731 |
|  | Distracted | 0.249  (0.234) | 0.122 (0.102) | P = 0.013 |

***Hosmer-Lemeshow Test and Goodness of Fit of the GP-MCHAT-R Model***

**Table S.4**

Hosmer-Lemeshow test results, N of groups=5

| Group | Prob | Obs. ASD | Exp. ASD | Obs. TD | Exp. TD | Total |
| --- | --- | --- | --- | --- | --- | --- |
| 1 | 0.0215 | 0 | 0.3 | 21 | 20.7 | 21 |
| 2 | 0.0718 | 1 | 1 | 20 | 20 | 21 |
| 3 | 0.2285 | 5 | 3 | 14 | 16 | 19 |
| 4 | 0.6256 | 7 | 9.4 | 14 | 11.6 | 21 |
| 5 | 0.8731 | 15 | 14.3 | 4 | 4.7 | 19 |

Hosmer-Lemeshow Chi2, df=3: 3.17

P-value=0.3667

**Table S.5**

Hosmer_Lemeshow test results, N of groups=10

| Group | Prob | Obs. ASD | Exp. ASD | Obs. TD | Exp. TD | Total |
| --- | --- | --- | --- | --- | --- | --- |
| 1 | 0.0158 | 0 | 0.1 | 14 | 13.9 | 14 |
| 2 | 0.0215 | 0 | 0.1 | 7 | 6.9 | 7 |
| 3 | 0.046 | 0 | 0.4 | 11 | 10.6 | 11 |
| 4 | 0.0718 | 1 | 0.6 | 9 | 9.4 | 10 |
| 5 | 0.1452 | 1 | 1 | 8 | 8 | 9 |
| 6 | 0.2285 | 4 | 2 | 6 | 8 | 10 |
| 7 | 0.4321 | 4 | 4.5 | 8 | 7.5 | 12 |
| 8 | 0.6256 | 3 | 4.9 | 6 | 4.1 | 9 |
| 9 | 0.7581 | 9 | 9.3 | 4 | 3.7 | 13 |
| 10 | 0.8731 | 6 | 5 | 0 | 1 | 6 |

Hosmer-Lemeshow Chi2, df=8: 6.63

P-value=0.5768

***Goodness of fit Graphs for the GP-MCHAT-R model***

**Figure S.1**

Histogram of predicted probabilities

**Figure S.2**

Predicted probabilities vs social15 values

***Model Consistency in Random Halves of the Sample***

To test the consistency of the GP-MCHAT-R model in different subsets of the data, we split the sample into two random halves (n1=46, n2=55). We used each model to produce the ROC curve in the estimation half-sample as well as in the other (validation) half-sample.

**Table S.6**

Models in random half-samples

|  | | **OR** | **P** | **95% Conf. Int.** | |
| --- | --- | --- | --- | --- | --- |
| **n=46** | Social-15 sec | 0.827 | 0.012 | 0.71 | 0.96 |
|  | MCHAT-R | 15.14 | 0.02 | 1.54 | 148.51 |
|  | constant | 0.521 | 0.605 | 0.04 | 6.18 |
|  | LR = -17.19 |  |  |  |  |
| **n=55** | Social-15 sec | 0.869 | 0.034 | 0.76 | 0.99 |
|  | MCHAT-R | 29.21 | 0.003 | 3.24 | 263.41 |
|  | constant | 0.228 | 0.247 | 0.02 | 2.79 |
|  | LR = -20.40 |  |  |  |  |

**Figure S.3**

ROC and AUC random halves of the sample

To test the applicability of the overall model to both half-samples we conducted a likelihood ratio test with the p-value=0.7933 indicating no significant difference between models.

To ensure the model applicability to different random subsamples of the data, we conducted an analysis to test the coefficient of the interaction terms between the dummy variable representing the two random subsamples with social15 and MCHAT-R in the model. The p-values for these interactions (0.681 and 0.622, respectively) suggest no difference in these coefficients when estimated in each subsample.

***Comparing the GP-MCHAT-R Model in the Age Groups Including Both Cases and Controls Against Other Ages***

We used a similar approach to what we used when testing random half-samples. We defined a dummy variable identifying the 45-49 age group vs other ages combined. Next we fitted a logistic regression model with social15, MCHAT-R, the age dummy variable and the interaction terms between the dummy variable and each of social15 and MCHAT-R. Testing the coefficient of the interaction terms is essentially the test the hypothesis that the coefficient of social15 and MCHAT-R are the same in both age groups. In our model the p-value for the interaction between the dummy variable and social15 was 0.781 and for 0.332, both indicating that the coefficient of the original model hold for both age groups.

***Graphs of LOWESS with lower smoothing bandwidth (bw=0.3) and actual proportions of gazes by Frame***

**Figure S.4**

Focus trends among the TD children and children with ASD in the 50-second video in visit 1 (LOWESS bandwidth=.3)

**Figure S.5**

Focus trends among the TD children and children with ASD in the 50-second video in visit 2 (LOWESS bandwidth=.3)

**Figure S.6**

Proportion of gaze by frame among the TD children and children with ASD in the 50-second video in visit 1


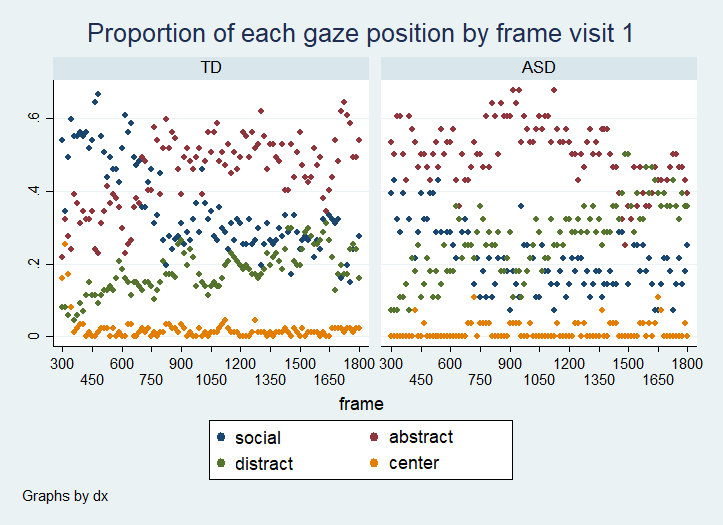


**Figure S.7**

Proportion of gaze by frame among the TD children and children with ASD in the 50-second video in visit 2


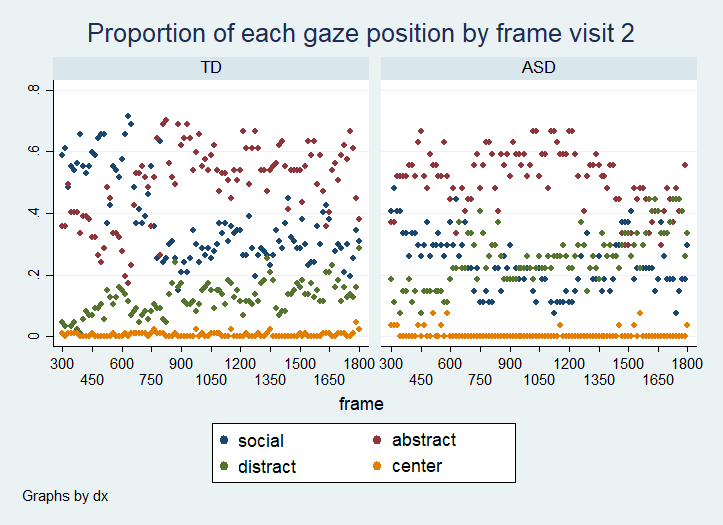

Supplement: Supplementary file 1 — Supplementary file1 (docx 222 KB) [file 10803_2021_4878_MOESM1_ESM.docx]
